# Beyond Statistical Significance: Implications of Network Structure on Neuronal Activity

**DOI:** 10.1371/journal.pcbi.1002311

**Published:** 2012-01-26

**Authors:** Ioannis Vlachos, Ad Aertsen, Arvind Kumar

**Affiliations:** Bernstein Center Freiburg, Neurobiology & Biophysics, Faculty of Biology, University of Freiburg, Freiburg, Germany; Indiana University, United States of America

## Abstract

It is a common and good practice in experimental sciences to assess the statistical significance of measured outcomes. For this, the probability of obtaining the actual results is estimated under the assumption of an appropriately chosen null-hypothesis. If this probability is smaller than some threshold, the results are deemed statistically significant and the researchers are content in having revealed, within their own experimental domain, a “surprising” anomaly, possibly indicative of a hitherto hidden fragment of the underlying “ground-truth”. What is often neglected, though, is the actual *importance* of these experimental outcomes for understanding the system under investigation. We illustrate this point by giving practical and intuitive examples from the field of systems neuroscience. Specifically, we use the notion of *embeddedness* to quantify the impact of a neuron's activity on its downstream neurons in the network. We show that the network response strongly depends on the embeddedness of stimulated neurons and that embeddedness is a key determinant of the importance of neuronal activity on local and downstream processing. We extrapolate these results to other fields in which networks are used as a theoretical framework.

## Introduction

*Nothing defines the function of a neuron more than its connections with other neurons* [1].

Systems neuroscience aims at gaining an understanding of how neural networks process information to implement specific functions in sensory, motor, and cognitive processing. To this end, the activities of multiple neurons are recorded simultaneously and analyzed to extract potentially relevant aspects about the task-related interactions among these neurons. If the analysis reveals statistically significant modulations of the recorded neuronal activity [2], then it is assumed that these spatio-temporal activity patterns are likely to play a role for processing and computation in the network.

However, the methods used to identify and measure the statistical significance of these patterns do actually not justify any claim regarding their *impact* on network dynamics or function. That is, statistical methods can demonstrate that a certain activity pattern appears beyond chance level or not. This in itself, however, does not suffice to stipulate that the recorded activity patterns are actually involved in processing or computation. In fact, in the following we argue that knowledge of the *statistical significance* of the recorded events is incomplete and needs to be complemented by additional information concerning the *structural and functional significance* of the neurons participating in these events.

## Simulation of a “Gedankenexperiment”

**Figure 1:**
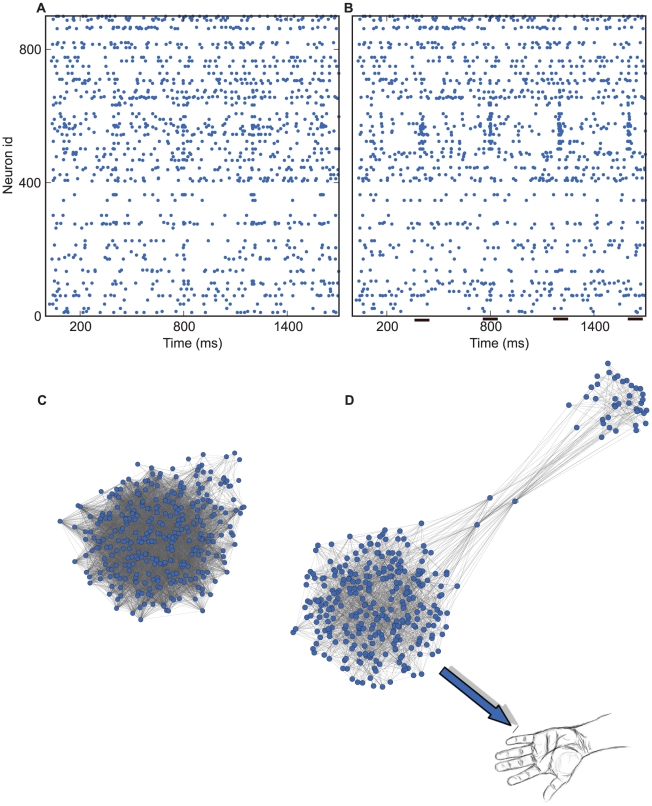
Statistically significant activity events in a modular network. (A) Rasterplot of excitatory (1–700) and inhibitory (701–900) neurons recorded in the simulation experiment. (B) Rows are sorted such that neurons with similar rate modulations appear together. Evidently, a subgroup of neurons fires action-potentials in a correlated manner during certain epochs in time (short black lines near bottom of the frame). (C) Schematic depiction of the underlying network from which neural activity was sampled. (D) The same network reorganized graphically using a force vector algorithm (cf. Methods) to reveal its modular structure. Note that in this Gedankenexperiment the big subnetwork controls the arm movement.

Let us consider a hypothetical experiment, in which neuronal activity is recorded from a certain brain area and the data is preprocessed to extract spike trains of 900 single neurons over a period of a few seconds (Figure 1A). The data are then analyzed to retrieve potential non-stationarities in the firing rates and correlations among the spikes of the recorded neurons. Indeed, in this simple example, 150 neurons out of the 900 recorded increased their firing rates in a correlated manner during short epochs of time (Figure 1B). Different statistical tests can be applied to demonstrate that the emergence of correlations among these neurons, during specific epochs, is indeed higher than expected by chance. However, this particular “Gedankenexperiment” enables us to go beyond merely establishing statistical significance of the activity modulations, by actually estimating the impact of these events on the brain area under consideration.

It is tempting, at first sight, to conclude that the statistically significant elevations of firing rates and increased correlations among the recorded neurons will have an impact on the dynamics and function of the network. To test whether this is justified, we investigated the topology of the network from which the spiking activity was recorded (Figure 1C). Indeed, having complete knowledge of the connectivity matrix allowed us to extract a graphical representation in which inter-connected neurons appear mutually closer in space (Figure 1D; cf. Methods). In this transformed representation it becomes evident that the network is in fact modular, consisting of two subnetworks, interconnected via a few nodes acting as hubs. Note here that vicinity in topological space does not imply actual physical vicinity. Relevantly, motifs and other ingredients necessary for such topological network arrangements have been identified in the brain [3]–[7].

The subpopulation of neurons exhibiting correlated activity in our example, in fact, stems from the smaller subnetwork. The transient increase in firing rates and correlation strengths during certain epochs is the result of a brief activation of the hubs that were designed to have strong uni-directional projections to the smaller subnetwork. Therefore, by construction, the activity of this subnetwork per se does not have any impact on the dynamics of the larger network or the hubs. Thus, knowledge of the network structure reveals that the observed statistically significant events are essentially an *epiphenomenon*, in the same way that the shadow of a moving person is an epiphenomenon of the movement; the observed events are the downstream result of the activation of some central nodes in the larger network, without these events themselves influencing the larger network at all.

Note that this is not meant to say that the activity of the small subnetwork is irrelevant or epiphenomenal in general. Rather, the message is that not all observed activity modulations of neurons in a task are relevant for the specific task itself, i.e., the subject's performance in the task and the neural computations underlying it (here, the task reduced to the desired hand movement required from the subject). Of course, the activity modulations in the small subnetwork could be relevant for some other aspect, not essential for the task itself—e.g., vision, memory, etc.

This observation has important implications for the understanding of the local network computations. If we assume, for example, that the larger network is part of an area in the motor cortex that controls a limb movement (Figure 1D), then investigating the dynamics of the smaller subnetwork would not be useful in any way to understand *how* the movement is encoded in the network, for the simple reason that the small subnetwork is not involved in the computations underlying the motor task. If, by contrast, it were the small subnetwork that controls the limb, then precisely this network should be investigated further, although, of course, it does not have any impact on the dynamics of the network it is embedded in.

In fact, the above scenario is not just a Gedankenexperiment. In human subjects performing a hand motor task, we recently observed that head movement was correlated with hand movement ([8]; S. Waldert, L. Tueshaus, A. Aertsen, C, Mehring, unpublished data). When the goal is to *decode* the hand movement from neural activity, then indeed the activity of the neurons encoding the head movement could be used for the decoding. However, when the goal is to *explain* the actual neural computations performed for executing the hand movement, then the activity of the motor neurons controlling the hand and not the head needs to be analyzed.

Another revealing example comes from studies by Riehle and colleagues investigating neural activity in the monkey motor cortex [9]. Specifically, they found that beyond the expected task-related motor responses, there were also neurons in the motor cortex that primarily responded to the visual cue in the motor task. Yet, they decided that, presumably, those responses did not primarily encode physical properties of the visual cue, but were, instead, involved in sensory-motor transformations [9]. That is, these stimulus-related events, although statistically significant, were “epiphenomenal” for visual processing.

These three examples clearly illustrate that statistical significance of recorded neural events is only a *necessary* but not *sufficient* condition for making inferences regarding the functional importance of these events for the computations performed by the investigated brain area. That is, knowledge of the way the recorded neurons are *embedded* in their local environment and of the structure of their projections onto downstream networks—denoted here by “structural significance”—is also important.

## Neuron Embeddedness

Here, we provide a formal definition of embeddedness. For this we distinguish between structural and effective embeddedness:

“Structural embeddedness” indicates the way neurons are physically embedded in their surrounding network. It can be characterized by graph-theoretical measures such as centrality, betweenness, k-shell index, etc.

“Effective embeddedness” is the influence neurons have on the activity of the surrounding network. Effective embeddedness is determined by structural embeddedness as well as by synaptic and cellular properties, ongoing activity, presence of neuromodulators, etc.

The concept of embeddedness has been initially used for socio-economic networks [10]. Within the context of systems neuroscience it extends the concepts of structural and effective connectivity by taking into account not only first-order but also all higher-order connections and neural interactions.

## Neuron Embeddedness and Population Response

The importance of the relative position of task-related neurons in the topological space of the network is not restricted to networks with a specific wiring. To test this, we performed a systematic analysis in which we investigated 100 different networks covering a wide range of topologies with variable characteristics ([11]; cf. Methods). To quantify the network topological properties, we calculated the small-world index (*SWI*) [12], [13] for all networks (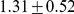; range 0.1 to 3).

All networks with *SWI* above unity were indicative of small-worldness. Small-world networks found in the brain have comparable *SWI* values [12]. Thus, to the extent to which *SWI* characterizes a network's topology, a high number of the model networks analyzed here (76 out of 100) had comparable topologies to those found in real brain networks.

**Figure 2:**
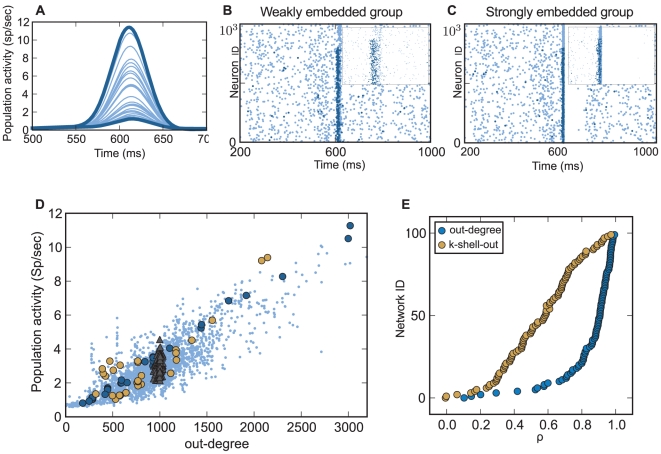
Structural embeddedness and population response. (A) Network response (PSTH) for identical stimulation of 30 different subpopulations of 250 neurons each (thin blue lines) in one example network. Observe that peak, onset, and rise times of responses of each subpopulation differ greatly. The thick blue lines depict the smallest and the biggest response, respectively. (B) Rasterplot of the network when the subpopulation of neurons with the lowest degree of embeddedness was stimulated. Light blue dots denote spikes from all neurons, dark blue dots those from stimulated ones. Inset: Magnified cut-out around 600 ms for neurons 4000–6000. Activation of weakly embedded neurons does not spread much in the network. (C) As in (B), but now the subpopulation with the highest average degree of embeddedness was stimulated, leading to a much bigger impact on the network activity. Activation of these strongly embedded neurons lead to a spreading of activity throughout the network. Moreover, feedforward inhibition suppressed the network activity entirely. (D) Response of all stimulated subpopulations (250 neurons each) and all networks pooled together (pale blue dots). On average, there was a positive correlation between out-degree and total network activity (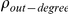 = 0.84). Two networks with small-world properties are highlighted (dark blue, light amber dots). The five random networks (filled gray triangles) did not exhibit high out-degree variance, and therefore the variance of their population response was small compared to that of the small-world networks. (E) Average correlation coefficient (sorted) between population response and degree of embeddedness as measured by out-degree and k-shell-out index. Both metrics had a high predictive power, with out-degree maintaining high prediction rates for most of the graphs. However, the predictive power of topology measures depended also on additional criteria (cf. main text and Figure 3).

For each network we performed multiple simulations, selectively applying a stimulus to a different subpopulation of 250 excitatory neurons to artificially render the correlations among them statistically significant. Subsequently, we estimated the effect of these statistically significant events on the entire network activity in terms of the peri-stimulus-time-histogram (PSTH) of the network activity (Figure 2A). Evidently, different groups of correlated events induced highly dissimilar responses in the network activity. For instance, there was a more than 10-fold difference between the weakest and the strongest response. Thus, although all events were statistically significant, their impact on the entire network differed substantially (Figure 2B and 2C).

This finding demonstrates that it matters which neurons in the network participate in the correlated events. In the networks used here, all stimulated neurons had identical intrinsic properties. Moreover, all their outgoing connections were of equal strength. Thus, the decisive factor determining the impact of a particular neuron on the overall network activity was the way it was embedded in the network. This degree of embeddedness of a node in the network can be quantified by different metrics from graph theory [14], [15], including the out-degree and k-shell-out index used here (cf. Methods).

To investigate the relationship between out-degree and network activity, we computed for each network the population response as a function of the average out-degree of all stimulated groups and all networks pooled together (Figure 2D). We found that, for any given network, the population response was stronger when neurons with higher out-degree participated in a statistically significant event (see Figure 2D). On average, the out-degree of the stimulated neurons was highly correlated with their impact on the overall network activity (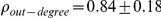).

Apart from the out-degree, however, other topological properties also affected the response. This is evident in cases where groups of neurons with comparable out-degrees had a quite different impact on the network activity (Figure 2D). Therefore, we also correlated the k-shell-out index of the stimulated neurons with the network response-strength (Figure 2E). We found that also the k-shell-out index was informative about the influence of a stimulated subgroup on the resulting population activity, albeit, generally, less than the out-degree (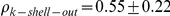; however, see below).

It may not be surprising that both the out-degree and the k-shell-out index of the stimulated neurons more or less adequately describe the neurons' impact on network activity. After all, both descriptors quantify the outreach of a neuron within the network. At the same time, our findings demonstrate that the combination of activated nodes (neurons) and topological properties of the network, irrespective of the method used to quantify them, do influence the network response and, therefore, should be considered in the analysis and interpretation of the recorded network activity.

### Interaction of Node Properties with Higher-Order Network Topology Descriptors

In the networks investigated here, we observed that the out-degree of a neuron was highly correlated with the impact this neuron had on the network activity. In this case, where neurons with regular-firing properties were used, the out-degree predicted a neuron's influence on the overall network dynamics quite well. However, in certain other, also biologically plausible scenarios, higher-order network metrics, such as the k-shell-out index mentioned above, could be a better estimator of neuron embeddedness.

**Figure 3:**
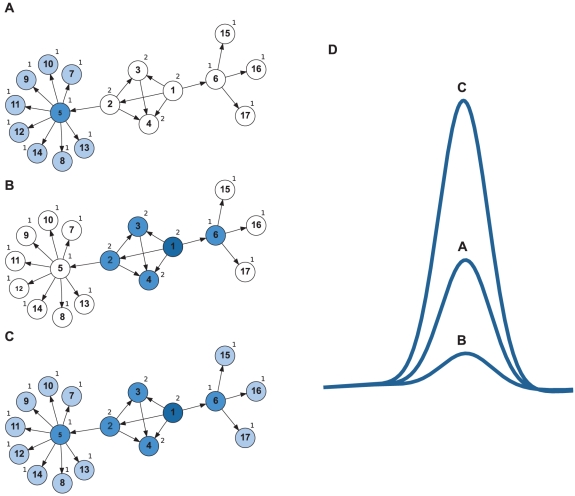
Interplay between node properties and higher-order network features. Example of a toy-network illustrating that the degree to which any given metric of neuron embeddedness predicts the neurons' impact on the population response may depend on single neuron properties. The small numbers next to each node indicate the corresponding k-shell-out index. (A,B) Neurons exhibited regular firing behavior. (A) A sufficiently strong input activating neuron 5 will yield propagation of activity to neurons 7–14. (B) If the same stimulus arrives in neuron 1, activity will only spread to neurons 2–4 and 6. In this case, the out-degree correctly predicts that the impact of neuron 5 is bigger than that of neuron 1. (C) Neurons exhibited bursting behavior. As previously, neuron 1 will activate neurons 2–4 and 6. However, the bursting response of these neurons may be sufficient to activate their post-synaptic targets as well, leading to spreading of activity over the entire network. Here, the impact of neuron 1 is clearly larger than that of neuron 5. This effect is not grasped by the widely used out-degree measure. However, higher-order network metrics, like the k-shell-out index, correctly assign a higher value to neuron 1, as compared to neuron 5. (D) Total network response in the three cases depicted in panels A–C. Note the higher impact of neuron 1 under some conditions (curve C), compared to that of neuron 5 (curve A).

We illustrate this scenario with a simple toy-network (Figure 3). Here, neuron 5 has a higher out-degree than neuron 1. That is, if neuron 5 is active, it will activate neurons 7–14 (Figure 3A). By contrast, neuron 1 will only activate neurons 2–4 and 6 (Figure 3B), thus having a smaller effect on the total network activity. However, if the neurons exhibit properties that facilitate spreading of activity, e.g., bursting behavior, then the activity of neuron 1 will first spread to neurons 2–4 and 6, and from there it will further propagate into the entire network (Figure 3C), whereas for neuron 5 such spreading will not occur.

In this example, simple out-degree-based methods would fail to predict the impact of a neuron. By contrast, the k-shell-out index would be more informative, because it is designed to address cases like the one illustrated here [16]. This suggests that the choice of the method to estimate neuron embeddedness should ideally incorporate knowledge concerning additional neuronal properties, such as their firing profile.

### Implications for the Interpretation of Neuronal Activity

One of the dominant approaches in systems neuroscience to understand the functioning of the brain is to record the activity of neurons under different stimulus and/or behavioral conditions, and to correlate the recorded activity with details of the task (stimuli, behavior). Indeed, since the seminal work of Adrian [17], Mountcastle [18], , Hubel and Wiesel [20], Barlow [21], Georgopoulos [22], etc., this approach has been successful in revealing neural correlates of various sensory, motor, and cognitive tasks, as well as in uncovering functional properties of neuronal networks in the brain. Recently, in the field of brain-machine interfaces, this approach has led to remarkable advances in decoding neuronal population activity [23], [24]. For these success, it was crucial to be able to demonstrate statistical significance of stimulus- or task-related neuronal activity. Thus, much emphasis has been given in devising appropriate null-hypotheses and performing adequate statistical tests [2].

However, successfully decoding neuronal activity does not imply an understanding of the actual computations performed by the underlying network. That is, statistical significance may be a *sufficient* condition for correctly decoding neuronal activity, but it is only a *necessary* one for understanding the computations performed by the network.

Here, we argue that an additional step towards unraveling the neural code, albeit not a sufficient one either as was elegantly demonstrated by Marom et al. [25], is to take into account the specific network topology of the investigated brain area. This knowledge may also provide a different perspective in the interpretation of the network activity. For instance, it is well known that when a stimulus is presented repeatedly, the variability of evoked cortical responses is often as large as the response itself. The origin of this large trial-by-trial variability has been suggested to be ongoing brain activity [26]. In our simulations, we observed that for the same input stimulus, the variability of the network response was strongly correlated with the embeddedness of the stimulated neurons. Thus, the high trial-by-trial variability in neural responses during the identical task could be partially explained by the activation, in each trial, of diverse subsets of neurons, with different degrees of embeddedness. Similarly, different degrees of “embeddedness” could also underly the highly variable behavioral responses elicited by single neuron stimulation in vivo [27].

Finally, we point out that calculated distributions, spectra, or various other measures of network activity, such as pairwise and higher-order correlations [28], information content [29], [30], frequencies of neural activity motifs [31], [32], precise spike patterns [33], unitary events [34], serial correlations [35], and population codes [36], should all be interpreted in light of the underlying network topology. Likewise, model-based data analysis methods such as generalized linear models [37] should also take the underlying network topology into account.

In addition, knowledge of network topology can be used to determine whether increased activity in a neuron is a consequence of local network activity or whether it is simply input driven. Furthermore, the stimulus response shown in Figure 2 could be tested for its statistical significance of the expected activity modulation, given a particular network topology.

Our results and their implications are not restricted to a particular measure of network response (here: population rate, measured by PSTH). Other descriptors of network activity, e.g., pairwise and higher-order correlations, would have led to similar conclusions. Although we examined a variety of network topologies, we used homogeneous synaptic weights and neuron properties for each network. Studying these properties in topologically diverse networks is an interesting endeavor in its own right and worth exploring further. For instance, as we have discussed above, the spiking behavior of neurons affects how well any specific measure of embeddedness predicts a neuron's impact on the network activity (Figure 3). Depending on these aspects, one measure of embeddedness may be preferable over another. Moreover, inhomogeneities in neuron and synapse properties may affect the embeddedness of a neuron per se, irrespective of the metric used. Thus, specific neuron properties could well modulate a neuron's impact on network activity.

In turn, the degree of embeddedness of any given neuron could restrict the impact specific neuron properties may have on the network. That is, although some neurons could exhibit “exotic” firing patterns, these may not have any effect on the network activity, if the associated neurons' embeddedness is low. This suggests that additional knowledge about single neuron properties becomes only meaningful once the degree of embeddedness of the neurons is known.

Embeddedness may be less important in classical random networks with a homogeneous topological space (Figure 2D, filled gray triangles) [38]. However, as soon as the topological space becomes inhomogeneous, it is vital to consider the structural properties of neurons and the networks they build. This is even more crucial for topologies in which the degree of embeddedness of neurons follows a heavy-tail distribution, such as in scale-free networks [39].

### Measures of Embeddedness

A number of properties of network connectivity have been shown to be important determinants for network activity dynamics [14], [15], [40]–[42]. Here, we used the out-degree and the k-shell-out index to predict the impact of stimulated neurons on overall network activity. We found that both metrics were correlated with the amplitude of the network response (Figure 2E); however, an exact prediction of this amplitude was not possible. In fact, it is very likely that multiple topology descriptors (e.g., betweenness centrality, eigenvalue centrality [14], [43]) may be both correlated amongst themselves and with the network response ([44]; S. Cardanobile, V. Pernice, M. Deger, S. Rotter, http://arxiv.org/abs/1112.3475). In fact, we found that betweenness centrality correlated well with the network response, at least for small networks (1,000 neurons; data not shown). That is, not any single metric, but rather a combination of different metrics might provide a better measure of embeddedness. Therefore, we need to extend previous work by defining a multi-dimensional descriptor of embeddedness, combining available measures with new ones that capture key features of network topology not considered thus far. In particular, there is a need for methods that can estimate neuron embeddedness from partial connectivity data to overcome the problem that the full connectivity matrix for neuronal networks [45] is not likely to be available in the near future.

Moreover, properties of individual neurons, e.g., those defining their firing patterns, may influence the effective connectivity in the network (Figure 3) and, thereby, affect the global network dynamics. In addition, synaptic properties—delays, time constants, type of neurotransmitter (excitatory or inhibitory)—and also ongoing network activity will contribute to the impact of a neuron on its embedding network. Hence, structural data on network topology, which only estimates “structural embeddedness”, need to be augmented by network activity data to obtain “effective embeddedness” of neurons.

We already mentioned k-shell decomposition as an example of a metric that goes beyond standard in- and out-degree measures. Other algorithms have been proposed to incorporate negative interactions between nodes [13], [43], [46], thereby rendering them more suitable for investigations of real brain networks. The inclusion of ongoing activity [26], stimulus-response relations [47], [48], response variability [49], and dynamic activity correlations [50], [51] will eventually lead to a dynamic measure of neurons' embeddedness.

This theoretical work needs to be paralleled by experimental approaches aiming at ways to measure the structural embeddedness of neurons in vivo. Evidently, knowledge of the full “connectome” [45] of the brain region in which activity is being recorded would be needed to ascertain the embeddedness of the neurons being recorded. In vivo measurement of the “connectome”, however, even of a small brain region, will not be feasible in the near future. Nevertheless, with existing methods it may be possible to indirectly estimate the embeddedness of neurons by selectively stimulating parts of the network and by measuring both extracellular and intracellular network responses to such stimuli.

In such experiments, modulation of extracellular activity (spikes and LFP) in a network would provide an estimate of the postsynaptic (suprathreshold) embeddedness of the stimulated neurons. In fact, such selective stimulation experiments would be similar to the ones we have shown and discussed in Figure 2. Similarly, measuring the subthreshold membrane potential of a neuron in response to stimulation of a subpopulation in the surrounding network could provide an estimate of the presynaptic (subthreshold) embeddedness of the intracellularly recorded neurons. Combining this approach with selectively visualizing the presynaptic neighbors of a given neuron [52], [53] might put the estimation of a neuron's embeddedness within reach. In addition, identifying the upstream or downstream connectivity [54] of recorded neurons will also contribute in estimating the neurons' embeddedness. An alternative approach has recently been applied to estimate the “structural” embeddedness of a neuron in vivo in its local microcircuit by juxtacellularly recording its activity and labeling it after the experiment [55].

In an ideal scenario, the brain area under examination could be scanned, before performing the actual experiment, to identify potential neurons to be recorded, based on their structural embeddedness. This would increase the chances of recording from those neurons that are involved in the local network computations in the investigated brain area. Alternatively, in an experiment where calcium imaging is possible, a wide array of stimuli could be used to obtain an average effective connectivity map of the area being recorded [5]. These and derived methods will also contribute in estimating embeddedness.

## Concluding Remarks

In neurophysiological experiments we see a continuing debate on the choice of appropriate null-hypotheses for testing the statistical significance of recorded spatiotemporal activity patterns [2], [31], [32], [56]. Adding another layer of complexity by estimating structural and effective embeddedness may appear to impede progress. However, as we have argued here, knowledge of embeddedness is indispensable to understand the functional role of neurons participating in statistically significant events.

To infer the function of networks in the brain from recorded activity of their member neurons, we need to differentiate between two issues: (1) how network structure and network activity affects a neuron's activity, and (2) how a neuron's activity affects network activity (and, perhaps, structure). The first of these two is increasingly becoming a research issue (see e.g., the Research Topic on “Structure, dynamics and function of brains: Exploring relations and constraints” in *Frontiers in Computational Neuroscience* [57]. Nevertheless, this increasing awareness has not (yet) influenced either the way data are typically analyzed or the way conclusions are drawn in large numbers of studies, in which recorded neuronal activity is primarily assessed for statistical significance.

Here, we argue that fulfilling statistical significance alone is not enough to stipulate a role of the recorded neurons in the computations performed by the network in the experimental task. This is precisely the point in the second issue mentioned above. It is here that we argue that structural and functional significance cannot be ignored. In fact, as our examples demonstrate, knowledge of the structural significance of the neurons participating in statistically significant activity events is indispensable. Thus, developing tools and methods to extract such information will in the long run facilitate our understanding of neural network functioning. This may eventually lead to the development of more appropriate null-hypotheses, where the statistical significance of expected activity modulations can be estimated, taking the network topology and its activity dynamics into account.

Finally, we emphasize that our results are not restricted to systems neuroscience. Rather, their implications permeate into every scientific discipline where networks are used as a conceptual and mathematical tool to examine and understand the observed activation phenomena. For instance, in epidemic research, the spread of diseases will be significantly influenced by the structural embeddedness of infected (humans) nodes. Here, the spread could be controlled by identifying and isolating highly embedded nodes, thereby removing the potentially high impact of these nodes on the evolution of the spread. Likewise, embeddedness could actually be used in controlling the dynamics of complex networks [58], and for other practical applications such as in controlling the spread of viruses in computer networks, of news and rumors in social networks, of power surges and load (im)balances in electricity networks and, turning now to clinical neuroscience, in efforts to regain control over pathological, uncontrollable neural networks (as in epilepsy and Parkinson's disease) by appropriate deep brain stimulation [59]. The further development of mathematical and experimental tools to estimate the embeddedness of network nodes will enhance our comprehension of various complex phenomena occurring in these types of networks [16], [60], [61].

## Methods

For the generation of the different network topologies, we used an in-house Python implementation of the multifractal network generator proposed by [11]. The graphical representation of the network in Figure 1 was designed using Gephi, an open-source graph visualization and analysis tool [62]. For the extraction of the modular structure of this network, we used the Gephi Force Atlas algorithm, a modified version of the Fruchterman-Reingold force-vector method [63].

The k-shell-out index of nodes in our networks was calculated by using the k-shell (also known as k-core) decomposition algorithm [16], [64]. The k-shell (or k-core) of a graph is the largest subgraph with minimum degree of “k”. The k-shell decomposition of a network involves systematically pruning it down to the nodes with k or more neighbors [16], [64], [65]. For the calculation of the small-world index (*SWI*), we computed the average shortest-path length 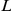 and the average cluster coefficient 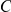 for each network. We normalized these values by the ones arising in the corresponding random network (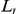 and 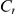, respectively). The corresponding random network was constructed with the Erdös-Rényi randomization model, which preserves the numbers of nodes, edges, and average connectivity, but not the specific network topology. The *SWI* is defined as the ratio of the two normalized metrics: 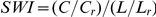. If 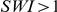, the network is said to exhibit small-world features.

The network simulations were performed with NEST [66]. Each network was composed of 8,000 excitatory and 2,000 inhibitory leaky-integrate-and-fire neurons with current-bases synapses. For each network, 30 different subpopulations were selected, each one with a different average degree of embeddedness as measured by the out-degree or k-shell-out index. In each simulation, stimulation was implemented by applying external Poisson input to all neurons in a subpopulation for 30 ms. The corresponding network response was measured by computing the peak of the population time histogram (Figure 2A).

## References

[1] Mesulam M (2005). Imaging connectivity in the human cerebral cortex: the next frontier?. Ann Neurol.

[2] Gruen S, Rotter S (2010). Analysis of parallel spike trains.

[3] Yoshimura Y, Dantzker JLM, Callaway EM (2005). Excitatory cortical neurons form fine-scale functional networks.. Nature.

[4] Song S, Sjöström PJ, Reigl M, Nelson S, Chklovskii DB (2005). Highly nonrandom features of synaptic connectivity in local cortical circuits.. PLoS Biol.

[5] Bonifazi P, Goldin M, Picardo M, Jorquera I, Cattani A (2009). Gabaergic hub neurons orchestrate synchrony in developing hippocampal networks.. Science.

[6] Yassin L, Benedetti BL, Jouhanneau JS, Wen JA, Poulet JF (2010). An embedded subnetwork of highly active neurons in the neocortex.. Neuron.

[7] Schnepel P, Nawrot M, Aertsen A, Boucsein C (2011). Distance and layer-dependent properties of horizontal projections onto layer 5 pyramidal neurons..

[8] Waldert S (2011). Cortical control of arm movement [PhD thesis].

[9] Riehle A (1991). Visually induced signal-locked neuronal activity changes in precentral motor areas of the monkey: hierarchical progression of signal processing.. Brain Res.

[10] Granovetter M (1985). Economic action and social structure: the problem of embeddedness.. Am J Sociol.

[11] Palla G, Lovász L, Vicsek T (2010). Multifractal network generator.. Proc Natl Acad Sci U S A.

[12] Bassett DS, Bullmore E (2006). Small-world brain networks.. Neuroscientist.

[13] Sporns O (2011). The non-random brain: efficiency, economy, and complex dynamics.. Front Comput Neurosci.

[14] Newman MEJ (2003). The structure and function of complex networks.. SIAM Rev Soc Ind Appl Math.

[15] Arenas A, Diaz-Guilera A, Kurths J, Moreno Y, Zhou C (2008). Synchronization in complex networks.. Phys Rep.

[16] Kitsak M, Gallos LK, Havlin S, Liljeros F, Muchnik L (2010). Identification of influential spreaders in complex networks.. Nat Phys.

[17] Adrian ED (1928). The basis of sensation.

[18] Mountcastle VB, Berman AL, Davies PW (1955). Topographic organization and modality representation in first somatic area of cat's cerebral cortex by method of single unit analysis.. Am J Physiol.

[19] Mountcastle VB (1957). Modality and topographic properties of single neurons of cat's somatic sensory cortex.. J Neurophysiol.

[20] Hubel DH, Wiesel TN (1962). Receptive fields, binocular interaction and functional architecture in the cat's visual cortex.. J Physiol.

[21] Barlow HB (1972). Single units and sensation: a neuron doctrine for perceptual psychology?. Perceptron.

[22] Georgopoulos AP, Kalaska JF, Caminiti R, Massey JT (1982). On the relations between the direction of two-dimensional arm movements and cell discharge in primate motor cortex.. J Neurosci.

[23] Donoghue JP (2002). Connecting cortex to machines: recent advances in brain interfaces.. Nat Neurosci.

[24] Schwartz AB (2004). Cortical neural prosthetics.. Annu Rev Neurosci.

[25] Marom S, Meir R, Braun E, Gal A, Kermany E (2009). On the precarious path of reverse neuro-engineering.. Front Comput Neurosci.

[26] Arieli A, Sterkin A, Grinvald A, Aertsen A (1996). Dynamics of ongoing activity: explanation of the larger variability in evoked cotical responses.. Science.

[27] Houweling AR, Brecht M (2008). Behavioural report of single neuron stimulation in somatosensory cortex.. Nature.

[28] Staude B, Rotter S, Grün S (2009). Cubic: cumulant based inference of higher-order correlations in massively parallel spike trains.. J Comput Neurosci.

[29] Rieke F, Warland D, van Steveninck R, Bialek W (1999). Spikes: exploring the neural code.

[30] Quiroga RQ, Panzeri S (2009). Extracting information from neuronal populations: information theory and decoding approaches.. Nat Rev Neurosci.

[31] Ikegaya Y, Aaron G, Cossart R, Aronov D, Lampl I (2004). Synfire chains and cortical songs: Temporal modules of cortical activity.. Science.

[32] Mokeichev A, Okun M, Barak O, Katz Y, Ben-Shahar O (2007). Stochastic emergence of repeating cortical motifs in spontaneous membrane potential uctuations in vivo.. Neuron.

[33] Abeles M (1991). Corticonics: Neural Circuits of the Cerebral Cortex.

[34] Riehle A, Grün S, Diesmann M, Aertsen A (1997). Spike synchronization and rate modulation differentially involved in motor cortical function.. Science.

[35] Farkhooi F, Strube-Bloss MF, Nawrot M (2009). Serial correlations in neural spike trains: experimental evidence, stochastic modeling and single neuron variability.. Phys Rev E Stat Nonlin Soft Matter Phys.

[36] Averbeck BB, Latham PE, Pouget A (2006). Neural correlations, population coding and computation.. Nat Rev Neurosci.

[37] Truccolo W, Eden UT, Fellows MR, Donoghue JP, Brown EN (2005). A point process framework for relating neural spiking activity to spiking history, neural ensemble, and extrinsic covariate effects.. J Neurophysiol.

[38] Erdös P, Rényi A (1959). On random graphs I.. Publicationes Mathematicae.

[39] Barabasi A, Albert R (1999). Emergence of scaling in random networks.. Science.

[40] Rajan K, Abbott LF (2006). Eigenvalue spectra of random matrices for neural networks.. Phys Rev Lett.

[41] Kriener B, Tetzlaff T, Aertsen A, Rotter S (2008). Correlations and population dynamics in cortical networks.. Neural Comput.

[42] Pernice V, Staude B, Cardanobile S, Rotter S (2011). How structure determines correlations in neuronal networks.. PLoS Comput Biol.

[43] Bonacich P (2007). Some unique properties of eigenvector centrality.. Soc Networks.

[44] Sporns O (2006). Small-world connectivity, motif composition, and complexity of fractal neuronal connections.. BioSystems.

[45] Seung HS (2009). Reading the book of memory: sparse sampling versus dense mapping of connectomes.. Neuron.

[46] Rubinov M, Sporns O, Thivierge JP, Breakspear M (2011). Neurobiologically realistic determinants of self-organized criticality in large networks of spiking neurons.. Plos Comput Biol.

[47] Boucsein C, Tetzlaff T, Meier R, Aertsen A, Naundorf B (2009). Dynamical response properties of neocortical neuron ensembles: multiplicative versus additive noise.. J Neurosci.

[48] Boucsein C, Nawrot M, Schnepel P, Aertsen A (2011). Beyond the cortical column: abundance and physiology of horizontal connections imply a strong role for inputs from the surround.. Front Neurosci.

[49] Nawrot MP, Schnepel P, Aertsen A, Boucsein C (2009). Precisely timed signal transmission in neocortical networks with reliable intermediate-range projections.. Front Neural Circuits.

[50] Aertsen A, Gerstein G, Habib M, Palm G (1989). Dynamics of neuronal firing correlation: modulation of “effective connectivity”.. J Neurophysiol.

[51] Aertsen A, Erb M, Palm G (1994). Dynamics of functional coupling in the cerebral cortex: an attempt at a model-based interpretation.. Physica D.

[52] Bock DD, Lee WCA, Kerlin AM, Andermann ML, Hood G (2011). Network anatomy and in vivo physiology of visual cortical neurons.. Nature.

[53] Rancz EA, Franks KM, Schwarz MK, Pichler B, Schaefer AT (2011). Transfection via wholecell recording in vivo: bridging single-cell physiology, genetics and connectomics.. Nat Neurosci.

[54] Komiyama T, Sato T, O'Connor D, Zhang Y, Huber D (2010). Learning-related fine-scale specificity imaged in motor cortex circuits of behaving mice.. Nature.

[55] Burgalossi A, Herfst L, Heimendahl MV, Förste H, Haskic K (2011). Microcircuits of functionally identified neurons in the rat medial entorhinal cortex.. Neuron.

[56] Roxin A, Hakim V, Brunel N (2008). The statistics of repeating patterns of cortical activity can be reproduced by a model network of stochastic binary neurons.. J Neurosci.

[57] Aertsen A, Rotter S, Kumar A, Cardanobile S (2010). Structure, dynamics and function of brains: exploring relations and constraints.. http://www.bcf.uni-freiburg.de/news/20110808-call-frontiers.

[58] Liu B, Chu T, Wang L, Zuo Z, Chen G (2011). Controllability of switching networks of multi-agent systems.. International Journal of Robust and Nonlinear Control.

[59] Benabid AL (2003). Deep brain stimulation for Parkinson's disease.. Curr Opin Neurobiol.

[60] Newman M (2002). Spread of epidemic disease on networks.. Phys Rev E Stat Nonlin Soft Matter Phys.

[61] Buldyrev SV, Parshani R, Paul G, Stanley HE, Havlin S (2010). Catastrophic cascade of failures in interdependent networks.. Nature.

[62] Bastian M, Heymann S, Jacomy M (2009). Gephi: an open source software for exploring and manipulating networks.. http://www.aaai.org/ocs/index.php/ICWSM/09/paper/view/154.

[63] Fruchterman TMJ, Reingold EM (1991). Graph drawing by force-directed placement.. Software Pract Exper.

[64] Seidman SB (1983). Network structure and minimum degree.. Soc Networks.

[65] Pittel B, Spencer J, Wormald N (1996). Sudden emergence of a giant k-core in a random graph.. J Comb Theory B.

[66] Gewaltig MO, Diesmann M (2007). NEST (neural simulation tool).. Scholarpedia.

